# Photobiomodulation in Patients Taking Denosumab: Case Report and Literature Review

**DOI:** 10.3390/dj13030128

**Published:** 2025-03-13

**Authors:** Gianluigi Caccianiga, Antonio Barbarisi, Paolo Caccianiga, Dorina Lauritano, Saverio Ceraulo

**Affiliations:** 1 Department of Translational Medicine, University of Ferrara, 44121 Ferrara, Italy; 2 Department of Medicine and Surgery, University of Milano-Bicocca, 20100 Monza, Italy; 3 Department of Biomedical, Surgical and Dental Sciences, School of Dentistry, University of Milan, 20122 Milan, Italy; 4 Fondazione IRCCS San Gerardo dei Tintori, 20900 Monza, Italy

**Keywords:** photobiomodulation, denosumab, MRONJ, external root resorption

## Abstract

**Background:** Denosumab is a human monoclonal antibody playing a central role in bone resorption. The impaired bone healing observed in patients on denosumab is linked to the drug’s inhibition of osteoclast activity. Photobiomodulation (PBM) has garnered attention as a potential adjunctive therapy for managing oral complications in patients on denosumab therapy. The aim of this study is to provide a review of the literature regarding the benefits of photobiomodulation therapy in patients taking denosumab while providing a case report of a patient treated with this therapy. **Materials and Methods:** Key terms were used to search PubMed (MEDLINE), Scopus, and Web of Science, and at last, 25 articles were compared. Following the proposed review, a case of a patient is illustrated. **Results:** Based on our literature findings, there are no papers regarding the benefits of photobiomodulation therapy in patients taking denosumab specifically, but there are articles regarding photobiomodulation therapy and MRONJ osteonecrosis patients, which can be caused by denosumab. **Discussion:** Despite all the limitations of the data in the literature, it can be deduced that there are evident benefits of photobiomodulation therapy in patients taking denosumab. The integration of laser-assisted techniques and photobiomodulation into MRONJ management protocols represents a significant evolution in treatment strategies. **Conclusions:** Further studies are needed to better understand a potential association between odontoclasts (which can cause external root resorption) and neoplastic disease or medication, as well as to explore the role of photobiomodulation in the therapeutic rehabilitation process.

## 1. Introduction

Denosumab is a human monoclonal antibody that inhibits the receptor activator of nuclear factor-kappa B ligand (RANKL), playing a central role in bone resorption [1]. It is widely used in clinical practice to treat conditions such as osteoporosis, bone metastases, and other diseases associated with bone loss. Although denosumab has shown significant efficacy in increasing bone mineral density and reducing fractures, it also has significant side effects, particularly on oral health. Patients undergoing denosumab therapy are at a higher risk of osteonecrosis of the jaw (ONJ), a severe condition that results in the death of bone tissue in the jaw, often following dental procedures such as extractions, implants, or other invasive treatments [1,2]. Osteonecrosis of the jaw can lead to pain, infections, and long-term oral complications, significantly affecting the quality of life of affected individuals.

The impaired bone healing observed in patients on denosumab is linked to the drug’s inhibition of osteoclast activity, which disrupts the normal bone remodeling process and hampers tissue repair after injury [3]. Photobiomodulation (PBM) has garnered attention as a potential adjunctive therapy for managing oral complications in patients on denosumab therapy. PBM, also referred to as low-level laser therapy (LLLT), involves the application of low-intensity light (red or near-infrared spectrum, 600–1000 nm) to tissues to stimulate cellular processes (such as collagen production, fibroblast proliferation, and increased blood circulation), including wound healing, pain reduction, and tissue regeneration [4].

PBM permits photophysical mechanisms that interact with cellular components, influencing mitochondrial activity and promoting tissue regeneration, making it a promising tool for precision medicine [5].

There is an improvement in the stimulation of the cytochrome c oxidase and increase in adenosine triphosphate ATP production that plays a key role in cellular energy production and regeneration, and there is an absorption of light energy by chromophores within cells, leading to biochemical changes that influence gene expression, cellular proliferation, and tissue repair, supporting its use in dentistry, dermatology, and musculoskeletal disorders [6,7].

Lasers have been introduced in orthodontics primarily due to their exceptional surgical precision and decontaminating properties, particularly in managing periodontitis, peri-implantitis, and implant stability [8,9]. Minimal laser surgeries often eliminate the need for sutures, ensure more comfortable post-operative recovery, and are especially well received by younger patients. Additionally, photobiomodulation (PBM) is employed to alleviate pain during orthodontic procedures [10], accelerate treatment duration, and enhance the quality and quantity of keratinized gingiva, which can frequently diminish during orthodontic treatments [11].

Photobiomodulation is also a versatile therapy widely used in medicine and dentistry, offering numerous applications without any associated side effects [12].

This is particularly relevant for patients receiving denosumab, who may have delayed healing responses after dental treatments; PBM has also been demonstrated to accelerate soft tissue healing by promoting collagen synthesis and improving blood flow to the affected area, which is crucial for post-operative recovery in patients who are at risk for delayed healing due to anti-resorptive therapy [13]. Furthermore, research has studied the role of PBM in enhancing bone regeneration and preventing complications like ONJ in patients undergoing anti-resorptive therapy, including denosumab, highlighting its potential to stimulate osteoblast activity and improve overall bone healing [14].

Photobiomodulatory therapy (PBM) with low-level laser (LLLT) treatment has demonstrated beneficial effects in the alveolar bone repair process, thanks to its ability to reduce pain and inflammation, as well as promote angiogenesis and accelerate the formation of a new bone matrix [15].

PBM could reduce the incidence of post-operative complications, including infection and delayed healing, in patients on bisphosphonates and denosumab who required dental extraction [16].

Studies emphasized the need for standardized protocols for PBM application in dental settings, particularly for patients undergoing treatments such as denosumab therapy, to ensure effective and consistent outcomes [17,18].

The aim of this study is to provide a review of the literature regarding the benefits of photobiomodulation therapy in patients taking denosumab while providing a case report of a patient treated with this therapy.

## 2. Materials and Methods

This review followed a precise search strategy.

The first step was to identify a set of key terms to be entered into the PubMed (MEDLINE), Scopus, and Web of Science search bars, the main databases for the medical and health sector. This approach enabled the use of precise terms to conduct a targeted survey. Boolean operators were then applied to create a search syntax to refine the topic selection as accurately as possible: “Photobiomodulation AND Denosumab”, “Photobiomodulation AND MRONJ”, and “Photobiomodulation AND Osteonecrosis”, ensuring that the articles examined met specific inclusion criteria. The initial filter involved selecting papers published in the last 15 years (2009–2024) to incorporate the most recent studies on the subject. Titles and abstracts were analyzed independently by two authors (A.B. and G.C.), and studies deemed inappropriate based on these criteria were excluded. Any disagreements were resolved by a third author (S.C.). The next step, in accordance with the predetermined eligibility criteria, was to select full-text articles in English on human subjects, discarding those that did not align with the study’s objective. The papers that met these criteria underwent further screening by the same two reviewers, with final decisions being made in consultation with the third author in case of discrepancies. Studies were considered admissible only if they defined a systematic search strategy, utilized internationally recognized databases for references, and analyzed data from descriptive studies, randomized clinical trials, or observational studies such as cross-sectional, case–control, and cohort studies. Exclusion criteria included in silico studies, non-human studies, inaccessible titles or abstracts, non-English articles, and papers lacking sufficient information on the review objective. After completing the research, the selected 25 articles were analyzed and compared. Following this review, a case report is presented: a 52-year-old patient diagnosed with non-Hodgkin lymphoma in March 2020, treated with denosumab until June 2021, and in clinical remission, who presented in January 2022 for observation with multiple root resorptions. The study was conducted according to the guidelines of the Ethics Committee of Comitato Etico Brianza and executed in conformity with the Declaration of Helsinki (Prevo, 6 May 2021). An informed and free consent form was submitted to the patient on the proposed treatment.

## 3. Results

**Figure 1:**
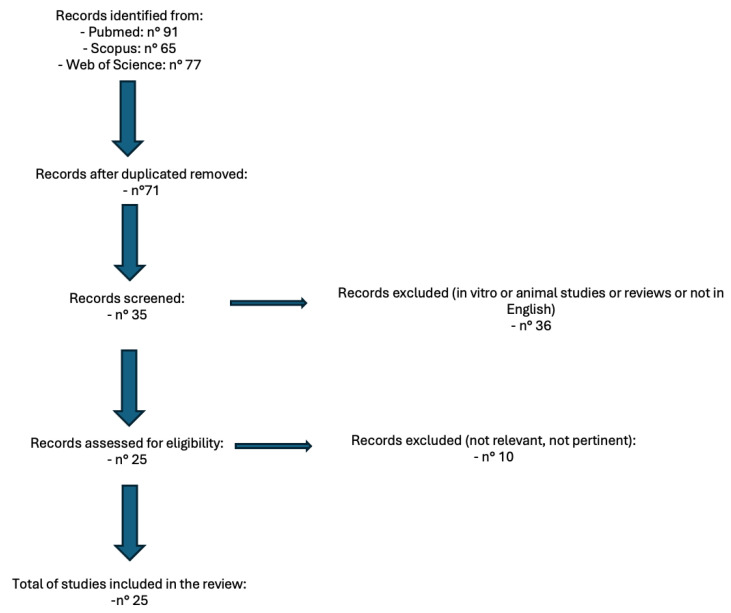
Workflow of article research.

This flow diagram below (Figure 1) illustrates the process of selecting articles for the review using “Photobiomodulation AND Denosumab”, “Photobiomodulation AND MRONJ”, “Photobiomodulation and Osteonecrosis”. The updated number of studies included is 25.

The articles selected are shown below (Table 1).

**Table 1:** Articles selected for this work.

N° Article	Article	Type of Study	Objectives	Conclusions
1	**Vescovi et al. (2024) [19]**	Case series	Evaluate the combined use of piezoelectric surgery, Er:YAG laser, and Nd:YAG laser photobiomodulation for MRONJ treatment.	The combined approach effectively treated MRONJ, improving outcomes and reducing morbidity.
2	**de Freitas et al. (2023) [20]**	Case report	Assess phototherapy and Er:YAG laser in managing mandibular osteoradionecrosis.	Phototherapy combined with Er:YAG laser promoted healing and reduced symptoms.
3	**El Mobadder et al. (2023) [21]**	Retrospective study	Investigate photobiomodulation with minimal surgical intervention for MRONJ.	Photobiomodulation is effective in enhancing healing with minimal surgery.
4	**Martins et al. (2021) [22]**	Case report	Evaluate photobiomodulation and antimicrobial photodynamic therapy for preventing MRONJ.	The preventive approach using phototherapy showed promising results.
5	**Monteiro et al. (2021) [23]**	Case report	Analyze LLLT effects on lenvatinib-related osteonecrosis of the jaw.	LLLT was effective in reducing symptoms and aiding recovery.
6	**Almeida et al. (2021) [24]**	Case report	Examine photodynamic therapy as an adjunct in MRONJ treatment.	Photodynamic therapy improved treatment outcomes as an adjunct therapy.
7	**Nica et al. (2021) [25]**	Case series	Combine photobiomodulation, surgery, and antibiotics for MRONJ management.	The multi-modal approach enhanced recovery and reduced complications.
8	**Torres et al. (2020) [26]**	Case report	Study LLLT as an adjuvant in MRONJ treatment.	LLLT effectively reduced pain and supported healing.
9	**Tenore et al. (2020) [27]**	Retrospective study	Investigate L-PRF and photobiomodulation in MRONJ management.	The combined therapy enhanced healing and patient outcomes.
10	**Magalhães et al. (2020) [28]**	Case report	Evaluate photobiomodulation and antimicrobial photodynamic therapy for osteoradionecrosis prevention.	The therapy demonstrated effectiveness in prevention and symptom control.
11	**Göl et al. (2020) [29]**	Comparative study	Compare extracorporeal shock-wave therapy and LLLT for bone healing.	LLLT was more effective in promoting bone healing.
12	**Tartaroti et al. (2020) [30]**	Case series	Analyze antimicrobial photodynamic therapy and photobiomodulation for MRONJ prevention.	Combination therapy effectively prevented MRONJ with long-term benefits.
13	**Fornaini et al. (2017) [31]**	Case report	Use laser and PRP to treat MRONJ.	Laser and PRP combination improved healing outcomes.
14	**Vescovi et al. (2015) [32]**	Case series	Develop a surgical protocol supported by LLLT for high-risk extractions in MRONJ patients.	The protocol reduced complications and promoted healing.
15	**Altay et al. (2014) [33]**	Retrospective analysis	Assess LLLT-supported surgical treatment of MRONJ.	LLLT improved surgical outcomes and reduced healing time.
16	**da Guarda et al. (2012) [34]**	Case report	Investigate GaAlAs laser photobiomodulation for bisphosphonate-induced osteonecrosis.	Photobiomodulation effectively managed symptoms and improved healing.
17	**Vescovi et al. (2012) [35]**	Retrospective analysis	Early surgical laser-assisted management of MRONJ.	Early intervention with laser therapy yielded positive long-term results.
18	**Martins et al. (2012) [36]**	Preliminary study	Examine laser phototherapy and PRP for MRONJ in cancer patients.	Combined therapy enhanced healing and reduced symptoms.
19	**Atalay et al. (2011) [37]**	Comparative study	Compare laser-assisted and conventional surgery for MRONJ.	Laser-assisted surgery showed superior outcomes.
20	**Luomanen et al. (2012) [38]**	Case report	Study Nd:YAG laser biostimulation for MRONJ treatment.	Laser biostimulation reduced symptoms and promoted healing.
21	**Romeo et al. (2011) [39]**	Preliminary study	Evaluate pain control in MRONJ with LLLT.	LLLT effectively reduced pain and improved quality of life.
22	**Manfredi et al. (2011) [40]**	Case series	Assess MRONJ management in osteoporosis patients.	Multi-modal approach showed effectiveness in managing MRONJ.
23	**Kan et al. (2011) [41]**	Case report	Study LLLT for tooth extractions in patients on zoledronate.	LLLT reduced complications and enhanced healing.
24	**Scoletta et al. (2010) [42]**	Prospective study	Investigate LLLT effects on bisphosphonate-induced MRONJ.	LLLT improved healing outcomes in preliminary results.
25	**Vescovi et al. (2010) [43]**	Case series	Evaluate surgical approaches with Er:YAG laser for MRONJ.	Laser-assisted surgery was effective in managing MRONJ.

### Clinical Case

**Figure 2:**
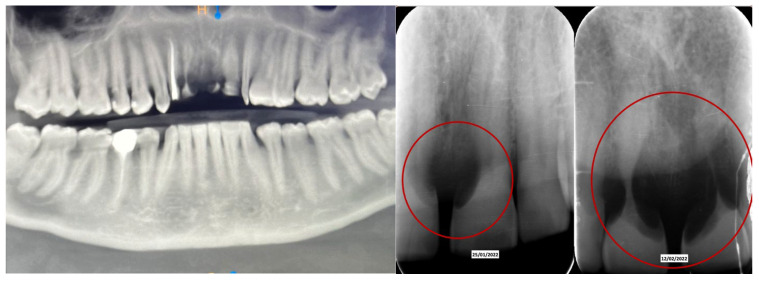
Evolution of the external resorptions, with a rapid deterioration in only 2 weeks.

A 52-year-old patient, affected by non-Hodgkin lymphoma since March 2020 and treated with denosumab until June 2021, in clinical remission, presented in January 2022 for observation with multiple root resorptions, not of endodontic origin; it initially affected 22-21-11, but then, with rapid evolution, also affected 23-12-13-14-15-16-17-46-47, without referred pain. The resorption phenomena ended in September 2022. Since then, every radiographic check-up carried out to verify the progress of the regenerative bone surgeries has confirmed the arrest of the progression of the resorption phenomenon affecting the residual dental roots. In February 2022, as soon as responsibility for the patient’s care was assumed, in agreement with the oncology department of the hospital in Bergamo in which he was in care, given the severity of the lesions present (Figure 2), it was decided to extract the teeth (22-21-11), with a surgical toilet of the alveolus associated with decontamination with dye-free laser photodynamic therapy (February 2022).

A single-stage photodynamic therapy session without dye involves several steps. First, there is irrigation with Sioxyl solution (Doctor Smile, Vicenza, Italy), a hydrogen peroxide derivative (stabilized H_2_O_2_ 10 vol. 3%). Then, the excess Sioxyl solution is aspirated while allowing some to remain inside the pocket or post-extraction socket for two minutes. Next, a high-frequency diode laser (980 nm) with a 400-micron fiber (Wiser Doctor Smile, Lambda, Vicenza, Italy) is inserted into the socket, reaching its depth. Finally, the subgingival tissues are exposed to laser radiation with a controlled back-and-forth motion using a dedicated program for 60 s per side, with a peak power of 2.5 W, a high frequency of 10 kHz, an average power of 0.5 W, and a fluency of 25,000/cm^2^.

**Figure 3:**
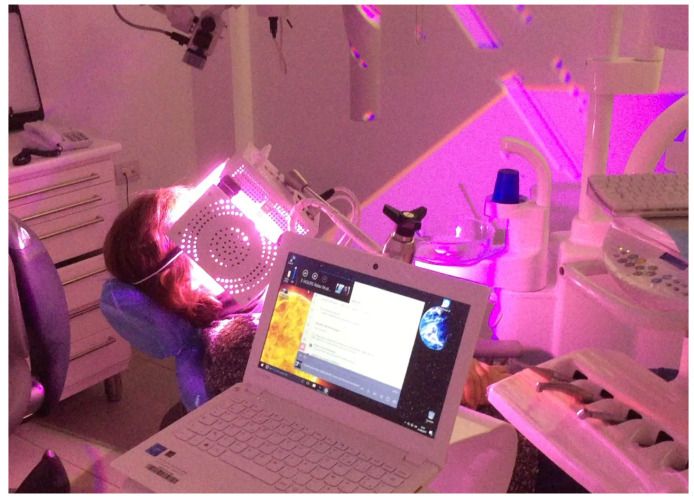
ATP38 device in a photobiomodulation session.

From that moment on, it was decided to subject the patient to fortnightly photobiomodulation sessions with ATP38 (Biotech Dental, Allée de Craponne, Salone-De-Provence, France), a device characterized by LED panels that mixes eight different wavelengths ranging from 450 to 835 nm (Figure 3), widely used for the treatment of oral mucositis following radio-/chemo-therapies. These LEDs are created with polychrome collimated semiconductors (PCSCs), which generate cold polychromatic light. This light promotes cell metabolism and stimulates the production of ATP (adenosine triphosphate, the main energy molecule of cells, also essential for DNA structure). 

The PBM session was selected based on the guidelines provided by the device. Each session delivers 6 min of irradiation with a fluency of 48 J/cm^2^, calculated by summing the fluency emitted by each cold light source—16 J/cm^2^—multiplied by the three distinct light groups (16 J/cm^2^ × 3 = 48 J/cm^2^). The fluency measurement was determined considering a 40 mm distance between the three light groups and the patient’s cheeks (lateral light groups) and lips (frontal light group). Each PBM session consisted of two consecutive irradiation phases, totaling 12 min and a fluency of 96 J/cm^2^. A 1 min relaxation period was included between the two stages.

**Figure 4:**
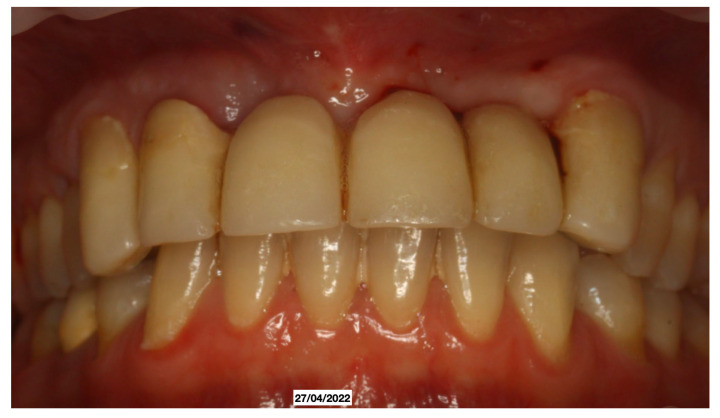
Fixed partial denture after preparing 23-12-13.

It was decided to temporarily rehabilitate the edentulism with a fixed partial denture after preparing 23-12-13 (Figure 4), keeping the adjacent teeth under observation but with a slight radiolucency already present.

**Figure 5:**
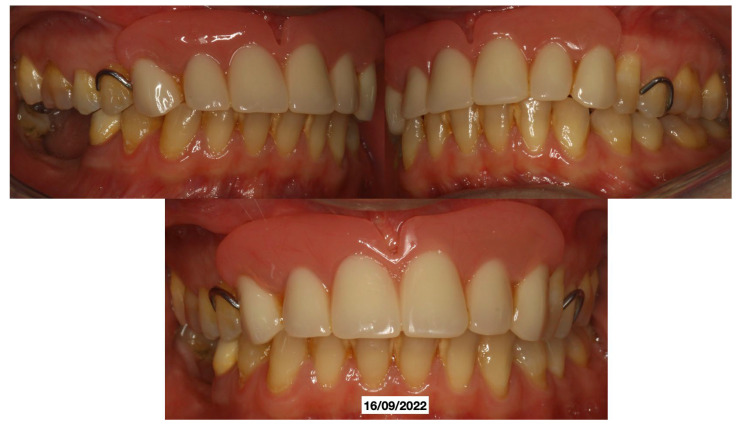
Removal partial prosthesis.

Just two weeks later, a rapid worsening was observed (documented with periapical intraoral X-rays) of the elements (13-12-23), which led to their necessary extraction (May 2022), with decontamination of the alveoli, with the beginning of a reabsorption process involving 14-15-16-46-47 (March 2022) with delivery of a removable partial prosthesis (Figure 5).

**Figure 6:**
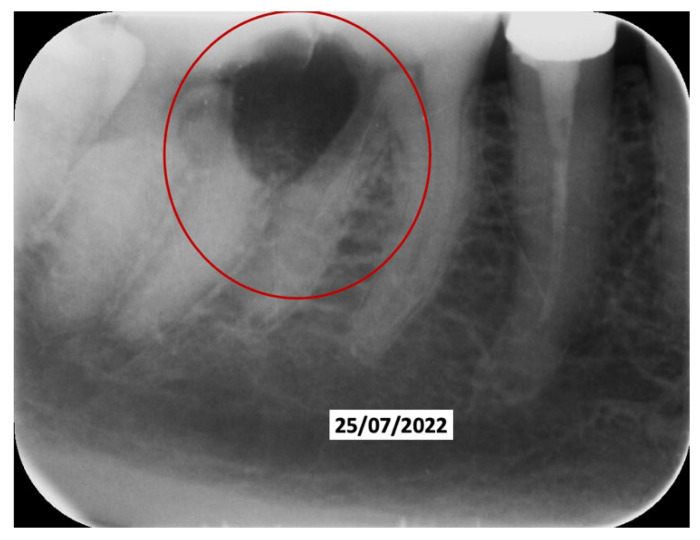
Evolution of the external resorptions in 46-47.

60 days after the extraction of the upper front teeth, it was discovered that the upper right premolars were also affected by significant root resorption, as well as 46 and 47 (Figure 6).

**Figure 7:**
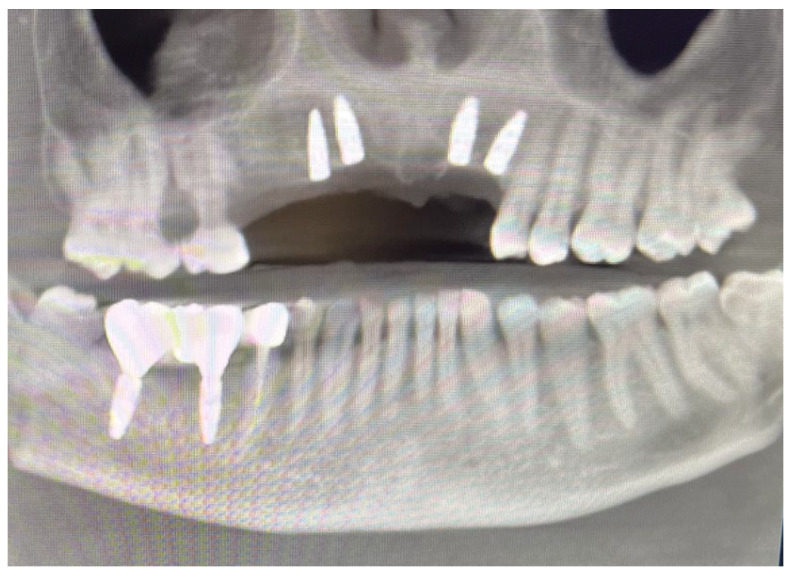
Ortopantomography (OPT) post-surgery in September 2023.

Based on previous experience and the speed of their development, in January 2023, it was decided to extract teeth 14 and 15, 46 and 47, decontaminating the alveoli, regenerating with PRF (in order to promote healing of the defect) and heterologous bone. So, the site was treated with a mix of Bio-Oss granules 25 (Geistilich Bio-Oss-Geistlich Pharma AG, Wolhusen, Switzerland), autologous bone chips, platelet-rich fibrin (PRF), and collagen membranes (45% Bio-Oss 25, 45% PRF, 10% autologous bone chips). Simultaneously, four implants (3.6 × 10 mm, Biotech Dental, Allée de Craponne, Salon de Provence, France) were inserted in the upper edentulous area, completely submerging them. However, an initial rejection of three of the four implants was observed (about 20 days after insertion), which made it necessary to remove them, decontaminate them with dye-free photodynamic therapy, and reinsert them 120 days later, when the implants were also inserted in areas 46-47 (3.6 × 10 mm and 4.2 × 10 mm, Biotech Dental, Allée de Craponne, Salon de Provence, France) in June 2023 (Figure 7).

**Figure 8:**
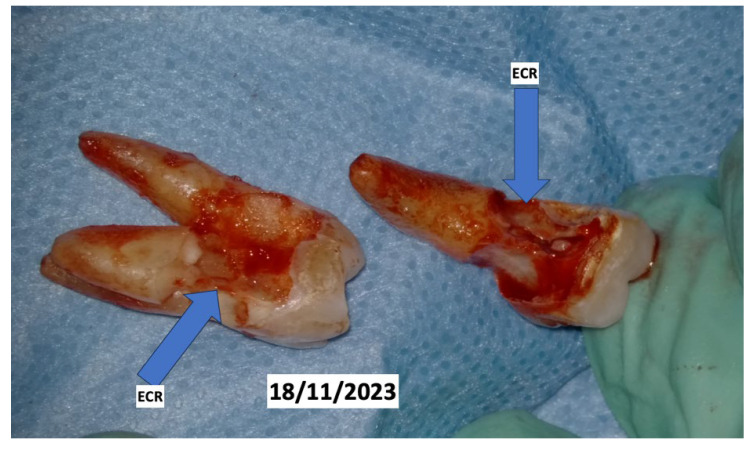
Teeth 16 and 17 extracted with an external cervical resorption (ECR).

In November 2023, teeth 16 and 17 were also extracted, always with the same surgical protocol of decontamination and photobiomodulation (Figure 8).

**Figure 9:**
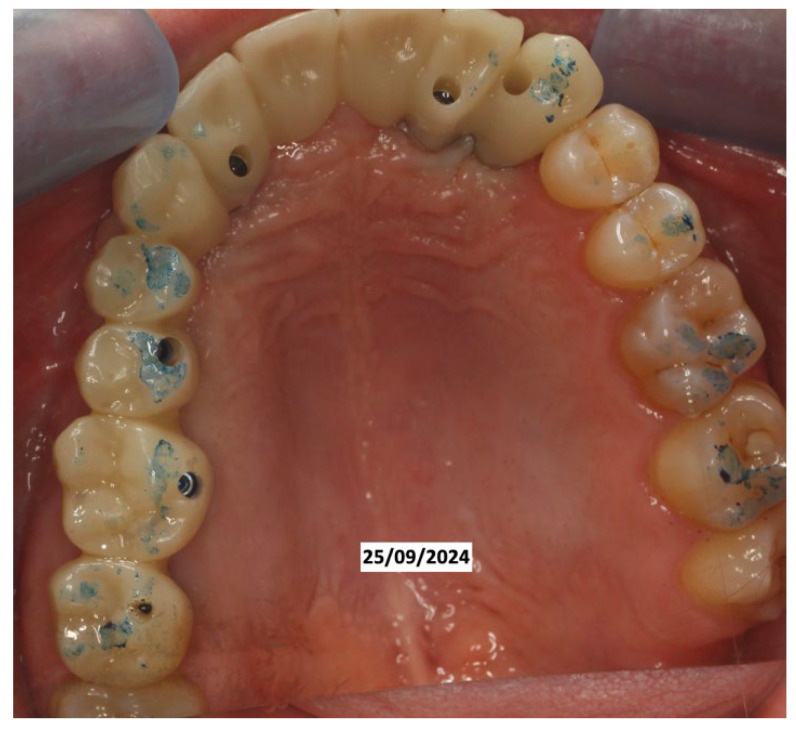
Fixed prosthetic rehabilitation.

**Figure 10:**
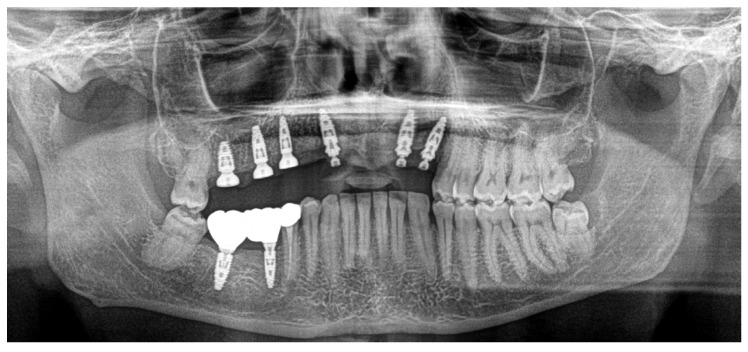
OPT after all implant placements, in July 2024.

In June 2024, the implants were inserted in areas 16 and 17. Three months after the insertion of the last implants in 16-17 (4.2 × 10 mm and 4.2 × 8 mm, Biotech Dental, Allée de Craponne, Salon de Provence, France), it was possible to proceed with the digital impression-taking to proceed with a temporary prosthesis in PMMA (Figure 9) after a radiographic control (Figure 10).

**Figure 11:**
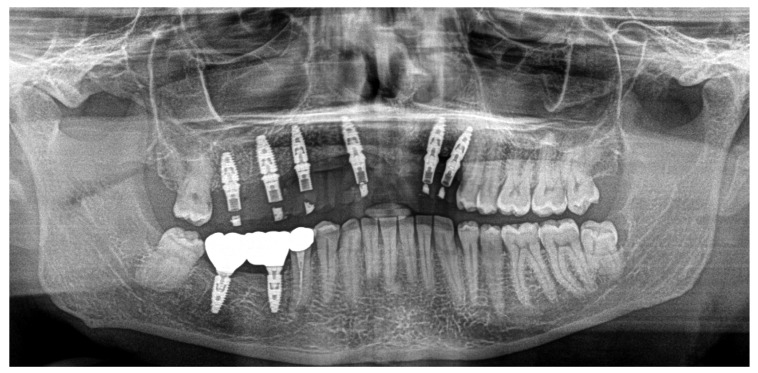
Radiographic follow-up in November 2024.

The case has now been finalized, and the radiographic follow-up shows a stability of the bone regenerations obtained (Figure 11), with no new external root resorptions on the residual teeth.

## 4. Discussion

Based on our literature findings, there are no papers regarding the benefits of photobiomodulation therapy in patients taking denosumab specifically in humans, but there are articles regarding photobiomodulation therapy and MRONJ osteonecrosis patients, which can be caused by denosumab. Some molecular mechanisms of interactions between PBM and RANKL/NF-kB are discussed in the literature, mainly in animal models. For example it is shown that low-level laser therapy (LLLT) can modulate the NF-κB signaling pathway in injured muscle tissue, reducing inflammation and promoting muscle recovery by influencing key molecular mediators of the inflammatory response and can activate NF-κB through the production of reactive oxygen species (ROS) in mouse embryonic fibroblasts, suggesting that laser-induced ROS generation plays a crucial role in cellular signaling and gene expression [44,45].

LLLT can also influence cell proliferation, apoptosis, and metabolic activity, with potential therapeutic applications in tissue regeneration and inflammatory modulation [46].

While our review provides a comprehensive overview of the available literature, the limitations in study design and quality of evidence must be acknowledged. Given the heterogeneity of interventions assessed, direct comparisons and definitive conclusions should be approached with caution. 

The management of medication-related osteonecrosis of the jaw (MRONJ) and related conditions has seen significant advancements through laser and phototherapy-based approaches. A combined method utilizing piezoelectric surgery and Er:YAG laser and Nd:YAG laser photobiomodulation demonstrated effective treatment outcomes, improving healing while reducing morbidity [19]. Similarly, phototherapy combined with the Er:YAG laser has been effective in managing osteoradionecrosis of the mandible, promoting healing, and alleviating symptoms [20].

Photobiomodulation (PBM) with minimal surgical intervention has been shown to enhance healing for MRONJ patients, underscoring its efficacy as a less invasive treatment modality [21]. Experimental studies have further supported the role of low-level laser therapy (LLLT) in preventing MRONJ-like lesions by promoting gingival wound healing through mechanisms such as IL-1RA-mediated pathways [32].

Preventive approaches, including photobiomodulation and antimicrobial photodynamic therapy, have yielded promising results in reducing the incidence of MRONJ, especially in high-risk scenarios [22,30]. Case reports highlight the benefits of LLLT in aiding recovery and symptom management for conditions such as lenvatinib-related osteonecrosis of the jaw [20] and bisphosphonate-induced osteonecrosis [34].

A multi-modal approach, integrating PBM, surgery, and antibiotics, has been effective in enhancing recovery and minimizing complications in MRONJ management [25,40]. Retrospective analyses and comparative studies consistently demonstrate that laser-assisted surgeries outperform conventional methods in terms of healing time and reduced complications [37,43].

Advanced therapies like laser phototherapy combined with platelet-rich plasma (PRP) have shown encouraging preliminary results, particularly for MRONJ in cancer patients [36]. Furthermore, studies focusing on pain control and quality of life improvements through LLLT have highlighted its role in comprehensive patient care [39,41].

In the clinical case described, based on the observations, it was found that the onset of external root resorption can be strongly associated with the patient’s clinical condition and the use of specific drugs, such as denosumab. This medication, by affecting the metabolism of bone cells, likely caused an “overactivation” of osteoclasts, leading to resorption not only of bone tissue but also of root structures, thereby potentially accelerating the progression of the pathology.

In the literature, we found a case report [47] describing a 74-year-old patient undergoing long-term treatment with denosumab for osteoporosis who developed aggressive external cervical root resorption (ECR) in multiple teeth, requiring extractions and prosthetic reconstruction. Denosumab effectively inhibits osteoclast activity by targeting the RANKL pathway, but the observed progression of ECR suggests the involvement of alternative, RANKL-independent pathways in odontoclastic resorption, highlighting a need for further research into the mechanisms underlying ECR and the potential side effects of prolonged antiresorptive therapy with denosumab, especially in patients with predisposing factors such as periodontitis.

Photobiomodulation, performed with ATP38 twice a month in the case report described in our article, as suggested by literature data [20,21,22], may have contributed to rebalancing osteoclastic functions, effectively promoting and consolidating bone regeneration and implant stability.

In fact, what we have observed is that by introducing photobiomodulation in the treatment, we have seen a clear improvement in the clinical picture. This could represent, albeit with the limitations of the few evidence present in the literature, an excellent therapeutic strategy.

The early failure of three out of the four implants placed could be attributed to the simultaneous extractions of teeth 14 and 15, which may have triggered a high systemic inflammatory index, preventing the integration of these implants, even though they were placed in bone regenerated with laser-assisted techniques.

Meticulous surgical cleaning of the post-extraction socket was also crucial, using dye-free photodynamic therapy for decontamination.

### Limitations

The research gap and future research ideas are shown in the table below (Table 2).

Further studies are needed to better understand a potential association between odontoclasts (which can cause external root resorption) and neoplastic disease or medication, as well as to explore the role of photobiomodulation in the therapeutic rehabilitation process.

## 5. Conclusions

Despite all of the limitations of the data in the literature, it can be deduced that there is the possibility of clinical benefits of photobiomodulation therapy in patients taking denosumab. The conclusions reached from our review and the illustrated clinical case cannot be definitive, but they need further studies to validate them. The integration of laser-assisted techniques and photobiomodulation into MRONJ management protocols could represent a significant evolution in treatment strategies. These methods not only improve clinical outcomes but also can reduce patient morbidity, emphasizing the importance of continued research and innovation in this field.

## Figures and Tables

**Table 2:** Limitations of the study.

Research Gap	Future Research Ideas
Poor statistical analysis to better demonstrate the benefits between photobiomodulation and denosumab	Experimental studies with the aim of establishing the objective parameters to evaluate the benefits of photobiomodulation on patients assuming denosumab
Data not sufficient to relate photobiomodulation effects on denosumab action	Experimental studies with the aim of establishing the impact of this therapy on the drug
Data not sufficient to relate denosumab action and involvement of odontoclasts, as shown in the clinical case	Experimental studies to better define this correlation

## Data Availability

Data from this study are available upon reasonable request by writing to the corresponding author.
